# Medical and Physical Intelligence

**Published:** 1805-05-01

**Authors:** 


### MEDICAL AND PHYSICAL INTELLIGENCE.

### [ FOREIGN AND DOMESTIC. ]

*Spontaneous Origin of the Rabies in Dogs, from a Communication of* Mr. HANNIBAL W. DOBBYN, *to* Dr. MITCHILL, *of New York, dated Janury* 26, 1802.

As I often reflect with attention on your doctrine of septon and on its effects, it appears to me that I shall be able to prove it to be the remote cause of rabies, hydrophobia, &c. in dogs. The following facts I adduce to support the assertion. In the autumn of the year 1782, a mania broke out amongst a pack of fox hounds which I had in Ireland, consisting of thirty couple; they were kept in a kennel, enclosed by a wall fourteen feet in height. I lost the whole of them by it, as I found that any fresh dogs brought from nurse, when put into it, were seized by the disorder, without being bit by any other animal whatsoever. I threw down the walls on two sides of the square, and had the rooms, boiler, vessels, &c. well washed with soap and water, and afterwards with vinegar; after which the place remained unoccupied for six months. I then brought home twenty-seven couple of whelps from nurse, amongst which I was not a little surprized to see the disorder begin to make its appearance in about a month after their arrival. I endeavoured to find out a cause in vain, for this disorder; it could not arise for want of a free circulation of air, as the walls were thrown down which might obstruct its progress; it could not proceed from the victuals, as I inspected them myself, and took proper care that they should be of the best sort, and that they should be well dressed and boiled. The back door of the stable opening into the kennel, the manure was thrown out into it: I observed that many of the dogs prefered sleeping amongst it, of a cold day or night, on account of the heat produced by its fermentation, to sleeping in the rooms of the kennel. Such of the hounds that did so, I observed were always seized with the distemper, and that it originated with them; and also that dogs, which lay in the litter with the horses in the stable, were seized with it, while those who slept in the haggard, under corn stacks, or amongst the hay, always escaped. From these observations I was certain that this distemper was occasioned by putrefaction, but how I was unable to comprehend. I endeavoured to ascertain the cause by dissection, and opened above a dozen of those which died of the distemper. Besides all the other symptoms common in the mania, I found the fleshy parts of the liver in a state of solution; in proportion to the progress of the disease, the larynx, &c most dreadfully constricted. May not the septic septic acid, taken up by those dogs from the dunghill and stable, be the cause of their madness, and, by dissolving the liver, the acrid effects of which operating on the larynx, cause that constriction which produces the hydrophobia? which can be, in my humble opinion, nothing else, but the highest degree possible of irritation of the nervous system, as a looking-glass kept in motion, before the eyes of a dog, produces the same effect on the system as water, into which, in a dark room, he will put his mouth, and endeavour to drink. And that those dogs were infected with the rabies, is confirmed by this proof, that all animals bit by them went mad (two cases excepted) in about fifteen days."

---

The following translation of a very flattering address in the German language to the Royal Jennerian Society, with Report of the Progress of the Jennerian Inoculation on the Confines of European Turkey, and the Report from India, have been handed to us by Dr. Walker.

The undersigned, late Province Physician at Varasden, in Croatia, in the years 1802 and 1803, (being encouraged by many successful examples, and supported by legal authority) inoculated above 1500 children, in his district, with cow-pock matter, received from Dr. De Carro, in Vienna, some of the children of high but most of low rank, and all with perfect success. Many of these were afterwards re-inoculated with the small-pox, without the smallest ill consequence, and he has had the pleasure of spreading the cow-pox, in the neighbouring countries of Hungary and Steyermark.

Penetrated with gratitude and the most profound respect towards Dr. Jenner, the Discoverer of Vaccine Inoculation, he thinks it his duty, and an honour, to communicate the intelligence thereof to the meritorious Royal Jennerian Society, in his own name, and that of every friend of humanity who has taken an interest in this heavenly benefaction.

MATTHEW GREGORIUS KRASKOVITZ,
*Feb.* 1, 1805. Practitioner at Vienna.

Report of Patients vaccinated by Mr. J. THORPE, Superintending Surgeon, in the Province of Guzurat and Surat, for the month of May, 1804. At Baroach, 113; Baroda, 200; Cambay, 112; Dholka, 70; Kairah, 20; Surat, 70; in camp, 221; total 806.

---

Mr. TAUNTON will resume his Summer Course of Lectures on Anatomy, Physiology, and Surgery, on Saturday, May 25, at eight o'clock in the evening, *precisely*, at the Finsbury Dispensary, St. John's Square. Practical Anatomy and Demonstrations in the forenoon as usual. Particulars may be known by applying to Mr. Taunton, No. 10, Pater-Noster Row, Cheapside.

---

Dr. JOHN REID is preparing for the press a work on the Nature, Progress, and Cure of Consumption, which will be published in the course of the autumn.

The Editors are sorry to have occasion to announce the decease of their Colleague Dr. Noehden, at Gottingen, a gentleman whose labours have so powerfully contributed to the reputation and great success of the Medical and Physical Journal. Arrangements are making, by means of which they hope to be able, in the next Number, to announce an able successor to, their lamented Colleague, in the Department of Foreign Correspondence and Intelligence.

